# Gain-of-Function Mutations in p53 in Cancer Invasiveness and Metastasis

**DOI:** 10.3390/ijms21041334

**Published:** 2020-02-17

**Authors:** Katarzyna A. Roszkowska, Slawomir Gizinski, Maria Sady, Zdzislaw Gajewski, Maciej B. Olszewski

**Affiliations:** Department of Large Animal Diseases and Clinic, Institute of Veterinary Medicine, Warsaw University of Life Sciences, 100 Nowoursynowska St., 02-797 Warsaw, Poland; katarzyna_roszkowska@sggw.pl (K.A.R.); slawomir_gizinski@sggw.pl (S.G.); m.sady92@gmail.com (M.S.)

**Keywords:** cancer, p53, gain-of-function, metastasis, invasiveness, review

## Abstract

Forty years of research has proven beyond any doubt that p53 is a key regulator of many aspects of cellular physiology. It is best known for its tumor suppressor function, but it is also a regulator of processes important for maintenance of homeostasis and stress response. Its activity is generally antiproliferative and when the cell is damaged beyond repair or intensely stressed the p53 protein contributes to apoptosis. Given its key role in preventing cancer it is no wonder that it is the most frequently mutated gene in human cancer. Surprisingly, a subset of missense mutations occurring in p53 (gain-of-function) cause it to lose its suppressor activity and acquire new functionalities that turn the tumor suppressor protein into an oncoprotein. A solid body of evidence exists demonstrating increased malignancy of cancers with mutated p53 in all aspects considered “hallmarks of cancer”. In this review, we summarize current findings concerning the cellular processes altered by gain-of-function mutations in p53 and their influence on cancer invasiveness and metastasis. We also present the variety of molecular mechanisms regulating these processes, including microRNA, direct transcriptional regulation, protein–protein interactions, and more.

## 1. Introduction

One of the results of the changes in demographic structure of many societies is an increasing prevalence of cancer. It is an extremely diverse disease, with many types of cancers originating in various organs. With progress in molecular diagnostics, cancers that previously have been thought of as a single disease are now known to have molecular subtypes that are predictive of their disease course and outcome. Being diverse as they are, most—if not all—cancers possess several features defined by Hanahan and Weinberg in 2000 as hallmarks of cancer [1,2]. These features that differentiate cancer from normal somatic cells are: self-sufficiency in growth signals, insensitivity to growth-inhibitory signals, evasion of apoptosis, limitless replicative potential, and tissue invasion and metastasis. Despite enormous progress in the field that occurred over these twenty years, these definitions are still valid, with an increasing body of detailed knowledge on molecular mechanisms underlying them. Most of these features are realized by cell-autonomous mechanisms; however, a solid body of evidence has been built demonstrating that cancer cells are able to tune their microenvironment to support their own growth, with induction of angiogenesis being one example. The involvement of mutant p53 in dysregulation of all of these features has been demonstrated [3]. This review is meant to briefly summarize the contribution of various p53 mutant proteins to cancer progression, with particular emphasis on mechanisms related to local tissue invasion and distant metastasis.

The *TP53* gene encodes p53 protein that, in its wild type form, acts as a tumor suppressor. In an active state, it is a homotetramer that binds to its specific target sites and modifies gene expression. Activity of p53 is crucial for control of cell cycle progression, apoptosis, cellular senescence, DNA repair, and genome maintenance. The crucial role of p53 in preventing cancer formation is underlined by the fact that, based on tens of thousands of records in International Agency for Research on Cancer (http://p53.iarc.fr) [4], over 50% of all tumors bear mutations in p53, more than in any other gene. Approximately 90% of these mutations are of the missense type and they are found in over 190 different codons; however, several of them are heavily overrepresented, with the eight most frequently occurring mutations in six different codons (R175, G245, R248, R249, R273, and R282) covering almost 28% of the patients [5]. These codon positions are termed “hot spot” and their mutated variants, particularly R175H and R273H, are by far best characterized in vitro, in vivo and in transgenic animal models. Amino acid changes in all of these positions disrupt p53 structure either globally (R175, R282) or locally, at the DNA-binding interface (positions 245–273), leading to the loss of p53 DNA binding and thus its function as a transcription factor. As p53 acts as a tetramer, even a single-allele mutation functionally inactivates the wild type allele by formation of heterotetrameric, transcriptionally inactive forms [6,7,8]; this mode of activity is referred to as dominant negative. The notion that mutated p53 may, in addition to losing its suppressor functions, also acquire new functionalities and thus act as an oncoprotein was formed in 1993 [9]; this mode of activity is referred to as gain-of-function (GOF). A number of studies on the roles of GOF p53 mutations in cancer physiology was performed using transgenic animals, cultures of primary tumor cells from such animals, as well as immortalized cell lines bearing GOF mutations in TP53 gene. In addition, in recent years, multi-thousand-patient molecular–clinical databases became available, allowing for discovery or verification of clinical phenotypes. These complementary approaches enable identification of molecular–clinical correlations in patient cohorts, creation of a transgenic animal model and dissection of molecular mechanisms underlying the clinical features in an in vitro organoid or cell culture model. Any potentially actionable molecular findings may then be sequentially tested using these models in order of increasing complexity. The “hallmarks of cancer” intertwine and have their partial contributions to various clinical manifestations of cancer patients. As the focus of the present review is on invasion and metastasis, other p53 GOF-related phenotypes will not be elaborated upon. Several excellent reviews were recently published on the subject [10,11,12,13]. To present the reader with the most reliable and reproducible effects of GOF mutations in p53, special attention was paid in this work to the best characterized and most frequently occurring variants, such as R175H and R273H. At the same time reports of novel and interesting insights resulting from expression of other mutated variants of p53 were included as well.

## 2. Animal Models of p53 GOF Mutations

Generation of p53 NULL mice in the early 1990s demonstrated in vivo that it is indeed a major tumor suppressor [14,15]. The first notion that mutated p53 may be increasing metastasis was made in 1988 by Pohl et al. [16], based on experiments with injections of genetically modified carcinoma cells. The observed oncogenic activity of mutated p53 was attributed to missense mutations and shown to differ between various p53 variants in a xenotransplant model of leukemic cells [17]. This study demonstrated that leukemic cells expressing R175H, R248Q, and R213Q p53 variants formed distant metastases, whereas the cells with Y234H and R273C variants did not. Moreover, the mice injected with metastasizing cells died more rapidly. The various GOF phenotypes of certain mutations in p53, including early tumor onset, changed tumor spectrum, and increased propensity for metastasis were demonstrated in numerous transgenic mouse models beginning in 2000s [18,19,20,21,22,23,24,25] as well as in clinical molecular databases [26,27].

Phenotypic variations of GOF p53 depend on the residue that is mutated as well as the amino acid that replaces the wild type variant. Although pooled patients with germline p53 missense mutations (Li-Fraumeni syndrome) do not develop the tumors earlier than the patients with NULL genotypes, early onset (7 years difference) is observed when the population of patients bearing R248Q (but not R248W) mutation is compared to NULL population. In addition, the distribution of primary tumor sites and the tumor spectrum in the large IARC cohort differs between patients bearing NULL and several “hot spot” mutated alleles [26,27]. Similarly, early tumor onset and shorter overall survival was observed in transgenic mice of *Trp53*-/R248Q and *Trp53*R248Q/R248Q genotypes when compared with their *Trp53*−/− counterparts [25], whereas the *Trp53*R248W/R248W mice presented more complex tumor spectrum than the *Trp53*−/− mice, but no significant change in survival [21]. In a murine p53-R172H model (equivalent to human R175H), the mice with *Trp53*+/R172H genotype exhibited marked increase in frequency of metastasis when compared with their *Trp53*+/− counterparts, but no decrease in overall survival was noted [18]. In another, independently created set of transgenic mice, also bearing R172H or R270H alleles (equivalent to human R175H and R273H, respectively) in WT or NULL backgrounds, the tumor spectrum differed, depending on the mutation, yet no significant change in the overall survival was noted. However, in agreement with results reported by Liu et al. [18], the mice with *Trp53*+/R172H genotype exhibited marked increase in frequency of metastasis when compared with their *Trp53*+/− counterparts [19]. Interestingly, no change in metastasis or survival was found in *Trp53*+/R172P mice [20]. In a bi-transgenic mouse in which endogenous p53 is functionally inactivated by constitutive expression of SV40 T-antigen and a mutated p53-R172H protein is expressed the former induces formation of mammary adenocarcinomas and the latter increases the rate of transition from intraepithelial neoplasma to invasive carcinoma. This leads to more severe tumor phenotype and increased rate of pulmonary metastasis [22]. In an inducible murine model of skin cancer the expression of p53-R172H in the background of active KRAS-G12D increased frequency of tumor formation, accelerated tumor growth and caused the appearance of metastases, when compared to p53-NULL variant [23]. Similar organ-specific expression of p53-R172H and active KRAS-G12D in murine pancreas yielded very aggressive and highly metastatic disease [24]. Due to limitations of rodent models, recently, several transgenic pig models were constructed. The first p53 knockout pig was created by targeting the locus with cre-inducible p53-R167H allele (porcine equivalent of human R175H). These animals prior to cre expression were effectively p53-NULL and following the activation accumulated high levels of mutated p53-R167H. They have developed spontaneous tumors, predominantly osteosarcomas, with primary locations and histological characteristics reminiscent of these found in humans [28,29]. These animals are a recent development and their lifespan is much longer than rodent’s and so full characterization of the tumor spectrum and differences between p53 NULL and R167H is still pending. Another transgenic pig combines expression of p53-R167H and KRAS-G12D. Local intramuscular injections of Cre-expressing adenovirus reproducibly generate mesenchymal tumors in these animals [30]. Although the cells isolated from these animals exhibit increased invasiveness in vitro and are tumorigenic in immunocompromised mice, the characterization of actual metastatic potential in physiological setting requires more time and effort.

## 3. Epithelial–Mesenchymal Transition and Related Phenotypes

For local invasion or distant metastasis to happen, tumor cells that are already immortalized still have to break several barriers. First, the cells have to acquire mobility and capability of extracellular matrix degradation. This, in conjunction with sensitivity to certain chemokines, enables directional migration through extracellular matrix. At this stage the tumor is capable of local invasion. If the cells can penetrate lymphatic or blood vessel wall, then they can travel to the local lymph node or a remote location, respectively. To survive in a blood or lymphatic vessel, the cells need to be resistant to anoikis (lose their anchorage dependence) and also evade immune cells. The organs targeted by metastases are non-random and that points to the fact that cancer cells exhibit a certain degree of tropism. That tropism results from sensitivity to certain chemokines and ability to adhere to the endothelium at specific locations. Such phenotypes are often connected to changes in expression of chemokine receptors and integrins. It is important to understand that a similar p53-induced change in phenotype may be caused by a number of molecular mechanisms and by a direct or indirect activity of mutated p53. These mechanisms include changes in miRNA, lncRNA, and interactions of p53 with transcription factors or chromatin. Conversely, a certain molecular mechanism may be involved in modification of various phenotypes.

In the process of spontaneous acquisition of metastatic properties, cancer cells often undergo epithelial–mesenchymal transition (EMT). This is a broad phenotypic change that allows the cells to gain mesenchymal properties such as ability to migrate or detach from surrounding cells. Although the changes in cells are dramatic, the whole process is controlled by a relatively small number of transcription factors such as SNAI1 (Snail), SNAI2 (Slug), TWIST1, and ZEB1/2 [31,32,33]. The reports that mutated p53 may be inducing EMT appeared nearly a decade ago [34], when p53-R175H has been shown to upregulate TWIST1, an EMT inducer, by reduction of its promoter methylation in immortalized prostate cancer cells. Subsequently, a correlation was demonstrated between mutations in p53 and changes in transcriptomic profiles of human breast cancers that were characteristic of EMT [35]. Another example of direct transcriptional regulation of EMT-controlling proteins in invasive breast carcinoma by mutated p53 is KLF17, a negative regulator of metastasis and EMT. Mutant p53 interacts with KLF17 and sequesters it, thus causing increased cellular mobility, decrease in apoptosis, and increase in chemoresistance. Some of the genes suppressed by KLF17 are *CD44*, *SERPINE1* (PAI-1) and *CCND1* (Cyclin D1) [36]. A more complex regulatory cascade was recently reported by Tanaka et al. [37], whereby mutated p53-G245D inhibited AMP-activated protein kinase thus decreasing the phosphorylation level of FOXO3a transcription factor and causing its cytoplasmic retention. This, in turn, led to derepression of FOXM1 gene, whose product is a potent EMT inducer [38]. Indeed, head and neck squamous cell carcinoma patients with mutated p53 and elevated FOXM1 expression showed significantly shorter survival [37]. An example of increase in both tumorigenic and metastatic traits resulting from mutated p53 expression was reported by Tan et al. In this work, the presence of p53-R273H (but not p53-R175H) increased AKT1 phosphorylation and suppressed BCL2 modifying factor (BMF) expression, thus increasing cancer cell resistance to apoptosis. This also applied to anoikis—detachment-induced apoptosis—therefore promoting metastatic phenotype [39]. Nearly 96% of patients suffering from high-grade serous ovarian cancer (HGSOC) have a mutation in p53 [40]. A mechanism involving small metabolite-based signaling was recently identified in a primary fallopian tube-derived cellular model of HGSOC [41]. In this model, p53-R273H reduced the expression of lysophosphatidic acid phosphatase type 6 (APA6), an enzyme responsible for degradation of lysophosphatidic acid (LPA) leading to its accumulation. High levels of LPA promote phosphorylation and activation of focal adhesion components FAK and paxillin and increased cellular motility. Indeed, in cells expressing p53-R273H increased number and size of focal adhesions was observed and in migration, invasion and xenograft transplantation models these cells exhibited increased malignancy and metastatic potential. Moreover, ovarian cancer and breast cancer patients with mutation in p53 had decreased levels of APA6 and ovarian cancer patients with low APA6 expression showed significantly shorter survival [41].

Following the reports that wild type and mutated p53 variants regulate several aspects of cancer initiation and progression through miRNA [42,43] further research demonstrated that this miRNA-mediated regulation also applies to EMT [44,45]. A simple example of this regulatory mechanism is an observation that expression of mutant p53-R175H in endometrial cancer cells decreased miR-130b levels by direct transcriptional repression, and thus led to derepression of ZEB1 and induction of EMT [46]. A much more complicated cascade was identified by Neilsen et al., whereby mutated p53 sequestered p63 causing derepression of miR-155 promoter. Increased levels of miR-155 led to post-transcriptional decrease in ZNF652, a zinc finger transcriptional repressor of key regulators of metastasis, and invasion: TGFB1, TGFB2, EGFR, SMAD2, and VIM [47]. Conversely, interaction of p53-R273H with p63 in H1299 lung cancer cell line was reported to decrease the expression of let-7i and thus induce its targets: E2F5, LIN28B, MYC, and NRAS, significantly increasing invasiveness and metastatic potential of the cells [48]. Also, some of the miRNAs identified in an in vitro differential expression regulation by p53-R273H screen were validated in clinical-molecular database (TCGA lung adenocarcinoma) and their levels found to correlate with lymph node metastasis (miR-132 and miR-147b) or with epithelial/mesenchymal characteristics of the NCI60 panel cells, as measured by E-cadherin/vimentin ratio (miR-194 and miR-378a) [49]. TGF-β paradoxically displays both tumor-suppressive and pro-oncogenic activities, depending on circumstances. A report by Ji et al. demonstrates that p53-R175H modifies signal transduction pathways in such way, that it attenuates the tumor-suppressive and activates the pro-oncogenic signaling. This is achieved by specific binding to SMAD3, but not SMAD2 protein, thus tipping the balance between the arms of TGF-β response, resulting in increased TGF-β-induced expression of Slug and multiple metalloproteinase genes and reduced TGF-β-induced p21 expression [50]. TGF-β signaling can also be modified by modifying receptor availability and activity. An interesting mechanism was reported by Vogiatzi et al., where various variants of mutated p53 caused Sp1-mediated increase in expression of endoplasmic reticulum ectonucleoside triphosphate diphosphohydrolase 5 (ENTPD5) that promotes correct folding of N-glycosylated membrane proteins. This increase was accompanied by an increase in in vitro invasiveness and in in vivo lung colonization assay. One of the proteins processed by ENTPD5 is endoglin (CD105), a TGF-β coreceptor and thus changes in this pathway resulting from mutated p53 expression may be responsible for observed phenotypes [51].

Mutations in p53 may lead to phenotypic changes that do not present full EMT profile or that are of different nature, yet still increase metastatic potential. These include sequestration and inactivation of p63 and p73 that has been observed in mouse models [52] and demonstrated to promote metastasis [53,54]. In a fat pad implant xenotransplant model, the invasive breast carcinoma MDA-MB-231 cells, endogenously expressing p53-R280K, showed contralateral lymph node positivity in 21 out of 22 animals. Following p53 silencing there were only 12 out of 22 animals with lymph node positivity. Also, following tail vein injection of MDA-MB-231 cells, there were 75–90 micrometastases found in each murine lung. Silencing of mutated p53 decreased this number to less than 20 micrometastases. Importantly, expression of p53-R175H in place of endogenous p53-R280K restored lung metastasis phenotype. Prometastatic phenotypes in cells expressing mutated p53 result from sequestration of p63, which has suppressive activity towards TGF-β-induced malignant responses [53]. Interestingly, p63 sequestration followed by promoter derepression is not the only possible mode of interaction. A case of recruitment of mutant p53 to p63-responsive elements in a promoter by p63, followed by an increase in transcription mediated by p53 transactivatory domain was reported. A number of mutated p53 variants, including R175H, R248Q, and R273H, were reported to interact with alpha-1 antitrypsin (A1AT) promoter, which contains p63 responsive elements, and to significantly increase the expression and secretion of A1AT protein thus increasing metastatic potential in lung cancer cells. This activity was not observed for wild type p53. In case of p53-R273L, a direct dependency of A1AT promoter binding on the presence of p63 was demonstrated [55]. In *Trp53*-/R172H cells derived from murine pancreatic ductal adenocarcinoma [24], a dependency of metastatic traits on expression of mutated p53 was noticed. It was found that in the p53-null cells the p73 protein was binding and sequestering NF-YB transcription factor and thus prevented expression of PDGFRB receptor. Upon mutation in p53, the GOF protein bound and inactivated p73 thus leading to the release of NF-YB and activation of PDGFRB expression. This, in turn, resulted in increase in sensitivity to microenvironmental cues and an increase in metastatic potential of the cancer cells [54]. In a similar murine model of pancreatic ductal adenocarcinoma significantly lower levels of miR-142 and more invasive tumor phenotypes were found in animals with p53-R172H mutation. It was established, that p53 GOF activity caused increase in DNMT1 (DNA methyltransferase) activity, increased methylation of DNA at miR-142 locus and thus decreased its expression; at the same time invasiveness of cancer cells was significantly increased. However, overexpression or miR-142 in the presence of p53-R172H decreased invasiveness to base levels, indicating that the increased malignancy was caused by p53-R172H/DNMT1/miR-142 cascade. Moreover, clinical database analysis demonstrated that both high DNMT1 and low miR-142 correlated with poor patient survival [56]. A novel mechanism for p53 GOF mutant action was identified in recent years that might provide even more diversity for its range of activities. In cells expressing several well-known GOF-mutated p53 variants (R175H, R273H, and R249Q), a smaller p53 isoform was observed. It was identified as Δ160p53 and its expression resulted from utilization of alternative translation start at codon M160. Some of the proliferative and metastatic traits observed in vitro were attributed to the presence of this isoform rather than full-length mutated p53, although the mechanism of action was not yet identified [57].

Another, relatively less known class of RNA molecules is long non-coding RNA (lncRNA). These molecules may serve as scaffolds for assembly of protein complexes. It has been recently discovered that two lncRNAs—lnc273-31 and lnc273-34—were upregulated in colorectal cancer cell culture model following the expression of p53-R273H. They are required for increased cell migration, proliferation and chemoresistance in vitro. In a murine xenotransplant model these lncRNAs dramatically accelerate tumorigenesis. Their expression is also significantly elevated in colorectal cancer patient tissues that bear R273H mutation in p53 [58].

Changes in cellular physiology induced by GOF mutations in p53 that manifest as clinically observed increase in metastasis may also concern changes in cytokine production and cytokine sensitivity as well as integrins and cytoskeleton organization. Although some of them are similar to what is happening during EMT, they may also occur as isolated phenotypes. It was reported that cancer cells expressing any of several mutated p53 variants (e.g., R175H, R248W, or R273H) released exosomes enriched in miR-1246 that were taken up by tumor-associated macrophages. This reprogrammed the macrophages into anti-inflammatory immunosuppression mode with increased release of TGF-β, which in turn supported tumor growth [59]. A case of p53-R273H-induced miRNA-based paracrine activity that eventually increased cancer cell mesenchymal phenotype was reported. Upon p53-R273H expression, HCT-116 cells released exosomes with increased amounts of miR-21 and miR-769. Levels of these miRNAs were also elevated in tumors with mutated p53, as compared to these with wild type p53. These exosomes were taken up by cocultured fibroblasts, activating SMAD/TGF-β pathway and causing increased release of this mediator, known for inducing EMT. Indeed, upon exposure to fibroblast-derived TGF-β there was an increase in expression of ZEB1 and SNAI2 and decrease in expression of several adhesion molecules in HCT116 cells [60], consistent with induction of EMT. Table 1 summarizes literature on EMT-related phenotypes regulated by mutated p53 as described in this section.

## 4. Inflammation, Chemokines, and Invasiveness

A link between inflammation and cancer has been established long ago and indeed inflammation is considered one of the “hallmarks of cancer” [1,2]. The topic is very broad and has been reviewed many times, also recently [61,62,63]. Similarly, involvement of wild type p53 in regulation of inflammatory response is well established [64,65,66]. In recent years, the role of p53 GOF mutations in regulation of cancer-related inflammation gained appreciation. It was demonstrated that these mutations can modify cancer cell reactivity to microenvironmental cues such as chemokines; they can also change cancer cell secretome thus altering the physiology of surrounding cells.

A tumor may be likened to a wound that never heals, thus inducing chronic inflammation. There are multiple ways in which tumor cells adapted to sustain and benefit from an ongoing inflammatory process. Due to the limited scope of the article the effects of loss of p53 activity will not be elaborated upon, but rather some examples of GOF p53 mutation-induced changes in the development of inflammation and their consequences for tumor invasion and metastasis will be presented. The key cellular regulator of inflammation is NF-κB and it was demonstrated in a number of in vitro and in vivo models that mutated p53 prolongs its activation in response to TNF-α and thus promotes chronic inflammation that eventually leads to tumorigenic transformation [67]. In glioblastoma patients GOF mutation in p53 correlates with clinically observed more intense inflammation and enhances expression of CCL2 and TNF-α in NF-κB-dependent manner. Release of NF-κB-dependent cytokines increases immune cells infiltration and reprograms the myeloid cells from M1-like tumor-suppressing phenotype to the M2-like tumor-promoting phenotype [68]. Chemokines produced by these cells can increase cancer cell oncogenic potential, including invasiveness [69]. However, mutated p53 not only potentiates TNF-α response, but also changes its profile. Di Minin and collaborators demonstrated that mutated p53 binds and inhibits DAB2IP tumor suppressor; this results in an increase in NF-κB and a decrease in ASK1/JNK activities in response to TNF-α stimulation and translates to changes in TNF-α-induced secretome towards more proinvasive and immunogenic [70]. Cells with mutated p53 release more factors that can promote migration and metastasis, such as CA12, MMP9, or CXCL10 [71,72]. Notably, a meta-analysis performed by Di Minin et al. demonstrated that the set of 10 genes most consistently differentially expressed in response to TNF-α in cells with mutated p53 is prognostic in triple-negative breast cancer patients with regard to disease-free survival. A direct interaction between mutated p53 and NF-κB in colon cancer cells was demonstrated and its functional consequences were investigated [73]. It was established that upon interaction the specificity of the p53/NF-κB complex changes and following TNF-α stimulation it binds a different set of gene enhancers, thus changing gene expression profile. Among the genes that were most upregulated in the presence of mutated p53 were MMP9, CCL2, and LTB. Importantly, invasion assays demonstrated that in the absence of TNF-α the expression of mutated p53 did not change cell invasiveness, but TNF-α-induced invasiveness critically depended on the presence of mutated p53. Work from Yeudall et al. performed in lung cancer cells identified CXCL5, CXCL8, and CXCL12 as transcriptional activation targets of mutated p53 via NF-κB, regardless of TNF-α stimulation [74]. These chemokines released by cancer cells expressing mutated p53 might increase cancer cell general motility in an autocrine manner thus supporting metastasis. Similar observations were reported by Zhang et al. [75], whereby in *Trp53*-/R172H mice elevated expression of PLAC8 (onzin) was found when compared to *Trp53*−/− counterparts; this resulted in an increase in metastatic traits in osteosarcoma cells, both in vivo and in vitro. The molecular mechanism involved increased expression of CXCL5 that, in an autocrine manner, enhanced ERK1/2 phosphorylation. Observed phenotypes were reproduced by addition of exogenous CXCL5 and they could be abolished by specific inhibition of ERK1/2. However, some phenotypic changes resulting from p53-R172H expression were not prevented by *Cxcl5* gene silencing, indicating that other mechanisms were also involved.

Several mechanisms related to mutated p53-induced changes in epigenetic gene expression regulation were proposed. In a transgenic mouse colon cancer model carrying deletions in Apc in a *Trp53*−/− or *Trp53*-/R270H background it was shown that while the *Trp53*−/− animals rarely developed invasive tumor phenotype, it was commonly found in *Trp53*-/R270H mice. The authors conclude that this might have been caused by an observed increase in chromatin accessibility resulting in upregulation of approximately 350 genes, many of which could be classified as proinflammatory [76]. In a report concerning mutated p53 impact on enhancer-regulated gene expression, Rahnamoun et al. reveal that following prolonged TNF-α exposure mutated p53 directly interacts with monomethyl transferase MLL4 and selectively promotes its enhancer binding. This leads to H3K4me1 monomethylation and p300 histone acetyltransferase recruitment that, in turn, performs H3K27ac acetylation. These modifications lead to increased enhancer activity and gene expression (e.g., MMP9 and CCL2). Depletion of MLL4 under these conditions led to approximately 70% decrease in invasiveness [77]. A mechanism of increased cellular receptivity was demonstrated in lung cancer cells expressing mutated p53 that showed increased motility and invasiveness. These cells also showed elevated expression of receptor tyrosine kinase AXL that has transforming and prometastatic activity [78]. The increase in AXL was independent of p53 transactivatory domain activity and was accompanied by increased acetylation of AXL promoter and increased binding of CREB, E2F, and EP300 in the promoter region. Phenotypes resulting from mutant p53 expression could be reversed by siRNA directed against AXL [79]. An indirect regulatory mechanism was demonstrated by Fontemaggi et al., who discovered that mutated p53 bound E2F1 and this complex activated transcription of ID4, a transcription regulator. ID4 bound and stabilized mRNAs for IL-8 (CXCL8) and GRO-α (CXCL1), which resulted in elevated expression of both these chemokines [80]. Interestingly, Yan and Chen reported that mutated p53 directly bound and activated CXCL1 promoter leading to increase in its expression, proposing an alternative non-exclusive regulatory mechanism [81]. Table 2 summarizes literature on phenotypes related to inflammation and chemokines regulated by mutated p53, as described in this section.

## 5. Metabolic Alterations and Invasiveness

The best characterized metabolic shift in cancer cells is known as Warburg effect [2]. In brief, cancer cells, even under physiological conditions, utilize glycolytic rather than oxidative pathway for their energy generation needs. The exact molecular mechanism of this metabolic change is not known, but it is controlled by oncogenes and tumor suppressors [82]. It was reported that wild type p53 repressed Warburg effect in cancer cells; moreover, introduction of GOF mutation in p53 caused further intensification of glycolysis. Mechanistically in the presence of mutated p53 a glucose transporter GLUT1 was translocated to the plasma membrane by activation of RHOA/ROCK signaling. The relation between Warburg effect and cancer invasiveness is often overlooked. Progressive acidification of local microenvironment increases tumor malignancy, including proliferation, chemoresistance and invasiveness [83,84]. Various types of cancer cells increase production of proteases upon increase in acidity; these include MMP2, MMP9, CTSB (cathepsin-B), and CTSL (cathepsin-L) [85,86,87].

## 6. Concluding Remarks

After forty years of scientific efforts directed at dissecting the role of p53 in tumor development and evolution an enormous amount of observations was made. An initial view that p53 was an oncoprotein was abandoned in favor of its tumor suppressor role. Several years later it turned out that under certain circumstances p53 may indeed become an oncoprotein. If one looks at the tumor development as a process of evolution and adaptation to hostile environment—patient organism—one cannot miss the high prevalence of certain mutations in p53. These overrepresented gain-of-function mutations were demonstrated to provide the tumor with numerous advantages in its struggle for survival and expansion. However, that also means that tumors rely on and are addicted to mutated p53 which potentially presents clinically actionable opportunities. This brief review presented a number of molecular mechanisms utilized by mutated p53 to exert its activities: epigenetic regulation, direct and indirect transcriptional control, miRNA-mediated control, and mRNA stability modification. Some of these act in a cell-autonomous manner and some in autocrine or paracrine manner. Certainly there are effects and modes of action that are unknown yet. However, due to the high prevalence of these mutations in cancer patients, every effort directed at identifying the molecular mechanisms of clinically relevant phenotypes may result in identification of actionable therapeutic targets.

## Figures and Tables

**Table 1:** Summary of literature on EMT-related phenotypes presented in the current work that are regulated by mutated p53, with proposed molecular mechanisms.

Reference	p53 Variant	Phenotype	Molecular Mechanism
(Godfrey et al., 2018) [56]	R172H	Invasiveness	miR-142 downregulation by Dnmt1-mediated DNA hypermethylation
(Kogan-Sakin et al., 2011) [34]	R175H	EMT induction	TWIST1 upregulation
(Ali et al., 2014) [36]	R175H	Increased cellular mobility, decreased apoptosis, chemoresistance	KLF17 sequestration
(Dong et al., 2013) [46]	R175H	EMT induction	miR-130b repression, (miR130b-ZEB1 axis)
(Ji et al., 2015) [50]	R175H	Activation of pro-oncogenic signaling	Selective Smad3 binding, Smad2/3 imbalance
(Weissmueller et al., 2014) [54]	R175H R272H	Metastasis	PDGFRB upregulation through p73/NF-Y
(Shakya et al., 2017) [55]	R175H, R248Q, R273H	Invasiveness	A1AT upregulation
(Cooks et al., 2018) [59]	R175H, R248W, R273H	Tumor growth	Macrophage reprograming by exosomes with miR-1246
(Tanaka et al., 2018) [37]	G245D	Invasiveness EMT induction	FOXO3a downregulation FOXM1 derepression
(Nielsen et al., 2013) [47]	R248Q R282W	Metastasis, invasiveness	p63 sequestration, (miR-155 upregulation)
Vogiatzi et al., 2016) [51]	R248W, R273H, R280K	Tumor progression and metastasis	Endoplasmic Reticulum UDPase ENTPD5 upregulation
(Tan et al., 2015) [39]	R273H	Cell survival and anoikis resistance	AKT-dependent suppression of BCL2-modifying factor (BMF)
(Chryplewicz et al., 2019) [41]	R273H	Metastasis, invasiveness, migration	APA6 downregulation, leading to LPA accumulation
(Subramanian et al., 2015) [48]	R273H	Metastasis, invasiveness	let-7i downregulation
(Datta et al., 2019) [49]	R273H	Metastasis	miR-132 and miR-147b
(Zhao et al., 2019) [58]	R273H	Cell migration, chemoresistance, proliferation	lnc273-31 and lnc273-34 upregulation
(Ju et al., 2019) [60]	R273H	EMT induction	Exosomes containing miR-21 and miR-769 increased TGF-β production in fibroblasts
(Adorno et al., 2009) [53]	R280K	Cell migration, invasiveness and metastasis	SMAD2/3, Ras (p63 inhibition)

**Table 2:** Summary of literature on inflammation- and chemokine-related phenotypes presented in the current work that regulated by mutated p53, with proposed molecular mechanisms.

Reference	p53 Variant	Phenotype	Molecular Mechanism
(Zhang et al., 2018) [75]	R172H	Metastasis	CXCL5-MAPK signaling pathway upregulation by PLAC8 (onzin)
(Yan & Chen, 2009) [81]	R175H	Chemoresistance	Direct CXCL1 upregulation
(Yeudall et al., 2012) [74]	R175H, R273H	Cell motility, metastasis	CXCL5, CXCL8 and CXCL12 upregulation by NF-κB
(Vaughan et al., 2012) [79]	R175H, R273H	Increased motility and invasiveness	Receptor protein tyrosine kinase AXL upregulation
(Di Minin et al., 2014) [70]	R175H, R273H, R280K	Invasiveness, inflammation	DAB2IP inhibition
(Fontemaggi et al., 2009) [80]	R175H, R273H, R280K	Neoangiogenesis	CXCL8 and CXCL1 upregulation by E2F1
(Cooks et al., 2013) [67]	R248W	Chronic inflammation- invasiveness, metastasis	NF-κB activation by TNF-α
(Rahnamoun et al., 2017) [73]	R273H	Cancer-promoting gene expression	NF-κB
(Rahnamoun et al., 2018) [77]	R273H	Invasiveness	MMP9 and CCL2 upregulation by MLL4

## Abbreviations

GOF	Gain-of-function
EMT	Epithelial–mesenchymal transition
